# Assistive Technology in Multiple Sclerosis Patients—Two Points of View

**DOI:** 10.3390/jcm11144068

**Published:** 2022-07-14

**Authors:** Agnieszka Korchut, Veronique Petit, Ewelina Szwedo-Brzozowska, Konrad Rejdak

**Affiliations:** Department of Neurology, Medical University of Lublin, 8 Jaczewskiego Str., 20059 Lublin, Poland; vpetit@onet.pl (V.P.); szwedo.ewelina@gmail.com (E.S.-B.); konradrejdak@umlub.pl (K.R.)

**Keywords:** multiple sclerosis, user requirements, robotic assistant, digital technology

## Abstract

Objective: The goal of our study was determining the current needs and acceptance of patients with multiple sclerosis (MS) in the field of assistive technologies using materials from the “RAMCIP” project (Robotic Assistant for Mild Cognitive Impairment Patient at Home). Methods: There were two target groups: a population with MS, and medical personnel experienced in treating MS patients. This study was based on a two-step design method (workshops and surveys). Using the Likert scale, we identified the prioritization of users’ needs. Additionally, demographic and disease-specific data and their correlations with each other and with the level of priority of functionality were analyzed. Moreover, the acceptance aspect of the assistant robot and the respondents’ readiness to use it were determined. Results: We gathered 307 completed surveys (176 from MS patients, 131 from medical personnel). Functional capabilities from the safety category were a high priority in most cases. The medium priority functions concerned daily activities that required physical assistance and home management. The differences in prioritization between the two groups were also found. Variables such as age, level of disability, cognitive impairment, depression, and fatigue were associated with the priority level of the functionalities. Conclusion: In summary, our findings might contribute to a better adaptation of robotic assistants to the needs and expectations of the MS population.

## 1. Introduction

Multiple sclerosis (MS) is a progressive neurologic disease clinically characterized by complex variable neurological condition that can affect motor, sensory, and autonomic systems [1]. MS is the third most common cause of disability among people between the ages of 15 and 50, with a mean age of onset at 32 years [2]. Multiple sclerosis does not normally decrease overall life expectancy, but severe disability is noted in 10 percent within 5 years, in 25 percent within 10 years, and in 50 percent within 18 years [3,4]. Limb movement impairment, fatigue, depression, and cognitive deficits are common in MS patients, and adversely affect the ability to perform many common daily activities. As the disease progresses, patients often experience a diminished quality of life, resulting from new impairments with accompanying limitations in activities, and restrictions in their lives. Progression of the disease increases the need for help with normal daily activities. Currently, many assistive technologies include robotic assistance, which are recommended to compensate for the functional limitations associated with MS. Assistive technologies are widely used worldwide by people with disabilities, and they are also relevant for MS patients [5]. Thanks to the advances in medical technology, patients can cope with their health problems and sustain self-care by having greater autonomy. Assistive technologies can satisfy the non-pharmacological needs of MS patients, which may result in increased self-esteem and a consequent improvement in the ability to perform daily activities. The basic advantages of the assistive technologies include that they are operated by the user, and are designed to be used to execute essential activities, such as cooking, taking medications, getting dressed, or participating in social engagements. Considering limitations of conventional interventions and clinical measures, digital technology has become increasingly used to capture the complexity of MS in clinical practice, observational studies, and clinical trials [6].

The overall aim of this study was to explore the needs and acceptance of patients with MS for assistive technology using material from the research project “RAMCIP” (Robotic Assistant for Mild Cognitive Impairment Patients at Home). RAMCIP is a domestic autonomously-moving service robot, with the aim to proactively and discreetly assist older adults with mild cognitive impairment (MCI) living independently at home. It is equipped with a built-in touch screen and a dexterous robotic hand and arm. The RAMCIP robot has high-level cognitive functions, enabling it to optimally decide when and how to assist, adjusting the scope of help to the user’s needs. The RAMCIP project was founded by European Programme Horizon 2020.

MS patients at various stages of their disease may face similar limitations as elderly patients with cognitive impairment; hence, the idea of getting the opinion on the needs of MS patients regarding assistive technology from two perspectives: the potential user and the medical staff. Currently, it is very important to deepen the knowledge about the needs of specific patient groups that can be satisfied by assistive technologies. It is worth mentioning that new technological solutions make it possible to improve the quality of medical and nursing care, and sometimes, they are the only option of help when face-to-face contact is impossible.

Digital technology offers hope for remote interventions both for rehabilitation (physical and cognitive) purposes and for improving overall clinical management, focusing on aspects that would otherwise be difficult to deal with in clinical practice [7].

The future of assistive robots will soon be linked to the need to support healthcare and nursing care. A better understanding of the needs of patients with multiple sclerosis provides valuable information on how to improve new technologies.

This paper also presents and correlates the demographic and disease-specific measures with functional requirements and their acceptance.

## 2. Materials and Methods

### 2.1. Participants

The first target group for this study were patients diagnosed with multiple sclerosis according to the revised McDonald criteria [8], aged ≥18 years with varying degrees of disease advancement and all MS subtypes. Participants were randomly recruited among those hospitalized in the Department of Neurology, Medical University of Lublin (LUM), or during routine medical visits in LUM in the years 2019–2020.

**Figure 1:**
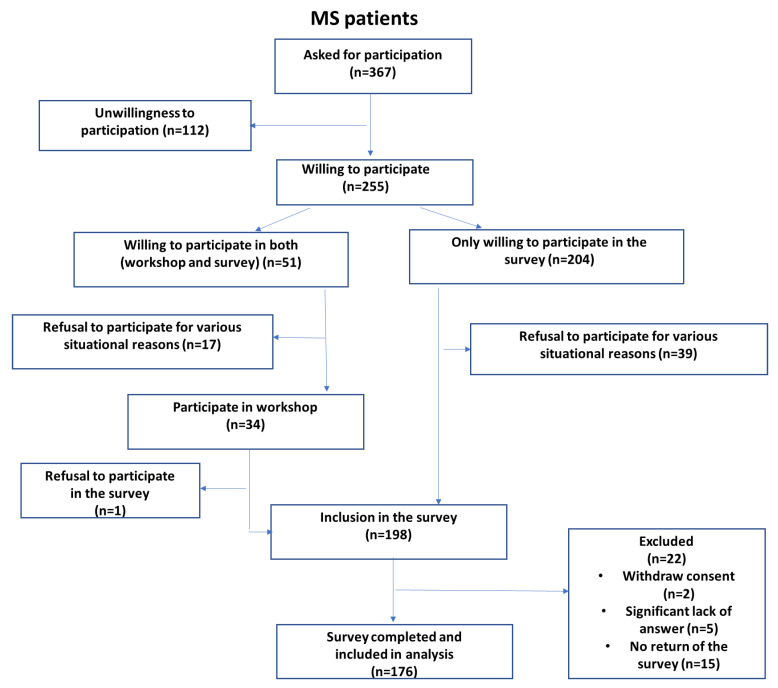
MS patients recruitment flow chart.

**Figure 2:**
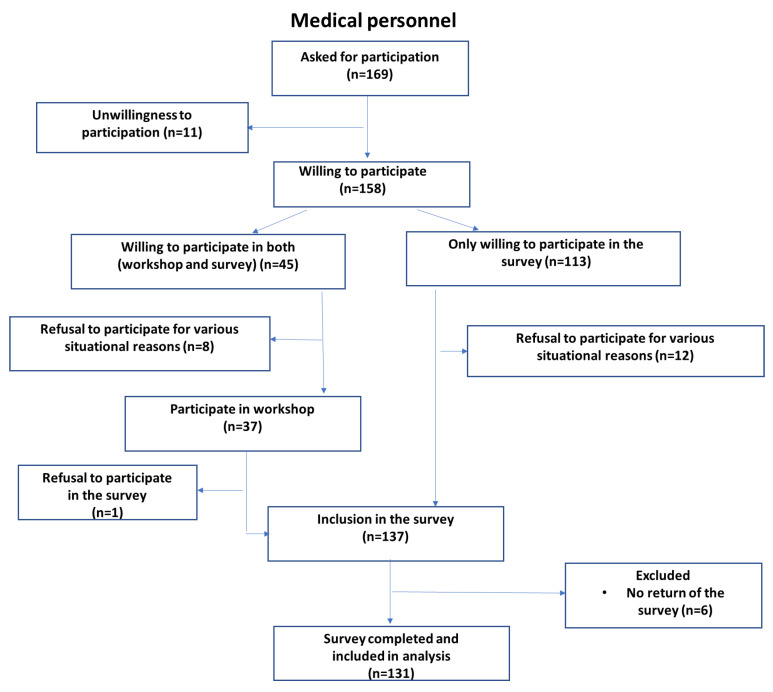
Medical personnel recruitment flow chart.

The second group was medical personnel with at least 3 years’ experience with MS patients. Figure 1 and Figure 2 show flow chart with details of the participant recruitment for the study.

### 2.2. Study Design

**Figure 3:**
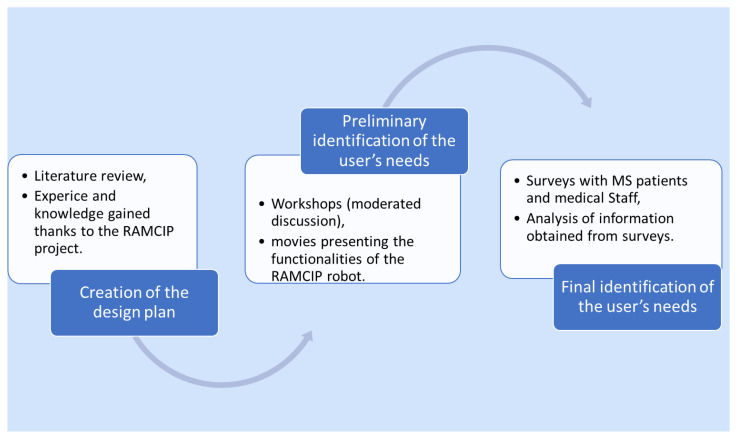
The study design flow chart.

In order to gather information on user needs related to assistive technology, the following steps were taken: creating a design plan based on a literature review, knowledge gained from the RAMCIP project, identifying initial user needs during the workshops, and defining the final needs of a patient with MS by analyzing survey data. Figure 3 summarizes the steps involved in the followed approach.

The workshops took place at the Department of Neurology of the Medical University of Lublin in five rounds: three times for MS patients in the number of 12, 12, and 10 participants; and twice for medical staff (20 and 17 participants). The workshops were led by a moderator (specialist in neurology).

During the workshops, film materials from the RAMCIP project were presented. The video shows the interaction between the RAMCIP robot and the user in a number of important aspects of the user’s daily life, from preparing meals, eating, taking medications, cognitive exercise, to ensuring user safety related to unexpected events, such as falls or misuse of household appliances. Participants shared their opinions and experience. In the next stage, the surveys were prepared. The layout of the surveys was constructed based on the experience gained during the implementation of the RAMCIP project. The survey scheme used in that project allowed for the collection of relevant data. Additionally, many of the questions were based on the ideas, knowledge, and experience of MS patients and medical personnel collected during workshops, as well as the literature review. The surveys for the MS group consisted of categories related to eight general issues: demographic data, disease-specific characteristics, neuropsychological status, nursing care requirements, usability of the RAMCIP system and other digital technologies, acceptance and readiness for use, human–robot interaction, and the design of the robotic assistant. With regard to the questionnaires for medical personnel, both had a similar layout, with the exception of three categories dedicated to patients with MS: disease-specific characteristics, neuropsychological status, and nursing care requirements. In the surveys, there were two kinds of questions: closed-ended and open-ended. Based on the answers from the closed-ended questions, we specified prioritization of the stakeholders’ requirements and needs regarding robotic assistants. Needs with high level (H) priority mean that a robotic assistant must have this function; medium level (M)—should have, if possible; and low level (L)—not necessary for implementation. Prioritization was performed using the Likert format of answers in surveys. It implies that the scores are valued as follows: 1 (very important, a patient has substantial difficulties with this, and the proposed solution is desirable) up to 5 (very unimportant, a patient can do this on their own without any difficulty, and the solution is not desirable) [9].

Questions about demographics and disease-specific characteristics were included in the surveys. To assess the level of severity of experiencing cognitive disability, fatigue, and depression, individuals were asked to rate the average level of severity over the last month on a numerical self-assessment scale from 0 (poorly) to 10 (very strong). The frequency of occurrence of the above-mentioned disorders was assessed from 0 (never) to 4 (almost always). The frequency criterion was specified as follows: almost never—less than 3 days a month, sometimes—3–15 days a month, often—more than 15 days a month, almost always—a maximum of three days without symptoms per month.

Regarding the assessment of cognitive functions, the patients were instructed what the cognitive functions mean, including learning, thinking, reasoning, remembering, problem solving, decision making, and attention.

Before completing the depression symptoms questionnaire, patients were asked to carry out the Beck Depression Inventory to better understand the symptoms of depression.

Disability levels were assessed using three categorized scores from minimal to advanced. Minimal indicated that individuals were able to walk without aid or rest for more than 500 m, intermediate were limited by their disability in daily activities, and advanced were restricted to wheelchairs. These categories are the self-administered version of the Expanded Disability Status Scale (EDSS). The category measures based on the EDSS were as follows: minimal (0–4.0), intermediate (4.5–6.5), and advanced (7.0–10.0) [5].

### 2.3. Statistical Analysis

Descriptive statistics were used to characterize the research participants. The presentation of collected data was dependent on the type of variables. Continuous variables were reported as means, standard deviation, and range, and categorical variables were presented as counts and percentages. The associations between demographic, disease-specific variables, and the results of the priority level of the functionalities were analyzed. The strength of these associations was explored using the Spearman’s rank correlation coefficient and Pearson correlation coefficient. All of the statistical analysis was performed using software Medcalc 12.2 (Ostend, Belgium).

## 3. Results

### 3.1. Profile of Participants

The total group of medical personnel consisted of 131 people (45 males and 86 females), including 39 doctors, 32 nurses, 32 physiotherapists, and 28 psychologists (mean age 38 ± 11 years). The mean value of experience working with patients suffering from MS was 11 ± 9.4 years.

Table 1 and Table 2 present the demographic and clinical characteristics of MS patients. The study involved 176 patients with MS (132 female, 44 male), nearly 90% of whom were in the 21–60 age range, and almost 63% of the respondents were economically inactive due to illness (80% 5 years after diagnosis).

Over 40% of the respondents reported moderate and severe functional limitations in tasks of self-care and household management.

More than 17% of the respondents required more than 10 h of assistance per week by a caregiver, in connection with the disease.

Over 30% of the respondents often or almost always reported cognitive problems, with the intensity level above 5 in 69%.

About 65% of the MS respondents declared using memory aids; in over 90% of the cases, they were smartphones with calendars, alarms, and a task list.

About 57% of the respondents stated that they often or almost always struggle with fatigue, 93% of which with a level of intensity above 5.

Approximately 22% of the respondents often or almost always reported depressive symptoms, with the intensity level above 5 in 95%.

### 3.2. Prioritizing Target Use Cases for the Robot Assistant

Functional abilities were divided into following categories: safety, cognitive aids, physical aids, household management, and socio-emotional wellness. Table 3 shows the priority functions of the robot assistant.

In most cases, no significant differences in prioritization between MS patients and medical personnel respondents were found. The categories related to user safety and physical assistance were the most extensive, with 11 and 8 topics, respectively. With regard to functionality related to user safety mentioned in the table, a high priority was calculated for 81.8% of target use cases for MS patients, and 90.9% for medical personnel. Only the issues related to the robot’s response to a fall or related to the opening of the door were rated as medium priority.

Regarding the part concerning physical assistance to the user, on the basis of the responses obtained from MS patients, a medium priority was calculated for 100% of target use cases, whereas for medical staff, it was 87.5%. Only stimulation to exercise and physical activity received high priority in the medical personnel group.

### 3.3. Prioritizing Human–Robot Interaction

As shown in Table 4, there are different capabilities of the robotic assistant in relation to human interaction. An analysis of 12 target methods of communication between the user and the robotic assistant was performed. Interactions related to the simplicity of the robot’s operation, such as reaction to simple commands and operation using a touch screen, were of high priority. Nearly all other ways of interacting were of medium priority.

### 3.4. Evaluation of the RAMCIP Appearance

Based on the percentage distribution of respondents’ answers, the RAMCIP appearance was assessed. The general appearance of the RAMCIP prototype reached a high-level attractiveness among two groups of the respondents (about 95%).

Most of them believe that the height of the robot assistant is appropriate, as it is lower than the user (90%). Others did not have an opinion on this subject.

Over 95% of the respondents from the two groups found the neutral appearance of RAMCIP highly appropriate. About 90% of MS respondents suggested that a robotic assistant should have a round face instead of a display showing an animated emotional facial expression. For about 60% of medical personnel respondents, it did not matter.

Additionally, MS respondents (over 50%) preferred more soft elements in the robot case compared to medical personnel respondents (about 20%).

### 3.5. Assistive Technology Acceptance and Readiness

The majority of the MS respondents (66%) shared the view that it is a good idea to replace a human caregiver with assistive technology. About 80% of medical staff respondents shared the same opinion, whereas the rest had doubts. Nurses had the most doubts, especially when they were over 50. Only two MS patients (1.14%) in the study would never agree to this type of solution.

In our survey, we found that 67% of MS respondents and 95% of medical staff respondents thought that the use of robotic assistance, such as that presented by RAMCIP, is more useful than any other type of application.

Approximately 66% of MS respondents agreed that they have no problem learning new technologies and applications, as well as that they enjoy learning new computer programs, technologies, and how to use new machines. About 16% of MS respondents reported that they had difficulty learning new technologies, and it was related to older age (above 60), higher levels of disability (at least 4.5 EDSS), and depression (level of intensity above 5) in the study population.

Readiness to cooperate with RAMCIP immediately was declared by over 19% of MS respondents; 10% believed that they would probably never agree to this type of cooperation; and the majority (over 70%) would like such a solution when they lose the ability to function independently.

About 90% of the participants agreed that RAMCIP is a useful and motivating technology. Nearly 90% of respondents strongly agreed that the RAMCIP presence would enhance the user’s security, and 83% agreed that it would benefit their quality of life.

### 3.6. Correlates of Demographics, Disease-Specific Variables, and Priority Level of the Functionalities

The level of importance of functionality, such as provision cognitive exercise, was associated with age, EDSS, and cognitive disabilities (*p* < 0.001). Endorsement of the absence of cognitive difficulties, lower levels of EDSS, and lower age were found to be associated with a lower level of significance of this functionality. Fatigue and depression (*p* < 0.001) were found to be associated with the level of importance of functionality related to the performance of physical exercises. Higher fatigue and the absence of depression were more likely to endorse a higher level of importance of this functionality. The data are shown in Table 5.

## 4. Discussion

This paper provides insight into acceptability and usability of the functionalities implemented to the RAMCIP, and presents priorities of desired task performance by a robotic assistant in the context of the MS population. The progression of MS is highly individual and unpredictable. A better understanding of the technological preferences of patients with MS is essential for the development of a robot intended to support the MS population. The rapid spread of the SARS-CoV-2 pandemic poses challenges to the management of persons with chronic diseases. One of the biggest challenges for new technologies dedicated to people with MS is finding a solution that meets the users’ needs and maintains or increases community participation with the consequent clinical benefits, cost-effectiveness, and user satisfaction. Specifically, we explored the needs of the MS population which could be fulfilled by a semi-autonomous system such as RAMCIP. User acceptance for semi-autonomous systems is higher than purely autonomous assistive systems [10].

The functionalities for monitoring and supervising the health and safety of the user at home are at a high level of priority. These include falls and the detection of dangerous home environment situations regarding working electrical or gas appliances left uncontrolled. A high level of priority also applies to the ability of a robotic assistant to inform the appropriate support units and relatives in the case of an emergency situation. Only functionality in the context of protecting the home against strangers was of medium priority in both study groups. Moreover, for respondents with MS, the ability of the robot assistant to ask about well-being after falls was a medium priority, whereas for medical staff, this skill was a high priority.

In a previous study which investigated prioritizing the requirements of a population with cognitive impairment, we found the same level of significance for most safety-related functions. Except for the difference in prioritization of the functionality related to the ability to recognize when a robotic assistant can or cannot open the house door, medical personnel in relation to the population with cognitive impairment reported that the robotic assistant must have this function (high priority) [11]. Over 90% of respondents strongly agreed that the RAMCIP presence would enhance the user’s security.

The most common source of home accidents is falls. Several symptoms of MS influence ambulation: loss of balance, weakness, fatigue, cognitive impairment, fear of falling, spasticity, tremor, and visual impairment [12]. Problems with gait affect 80% of patients with MS within 10–15 years of disease onset [13]. Thus, it is not surprising that falls are common in people with MS. Finlayson et al. reported that 52.2 percent of 1089 persons with MS aged 45 to 90 years had experienced a fall in the past 6 months [14]. Common sequelae of falls include compromised mobility due to physical injury, as well as mental trauma, such as loss of confidence in performing tasks and fear of falling. In this respect, the capabilities of a robotic assistant affecting the prevention of falls unanimously obtained high priority in the two study groups.

The tasks of reminding about and monitoring medication intake were assigned as high priority functionalities which must be implemented into the robotic assistant. Incorrect intake of medication is an important healthcare problem that generates avoidable costs [15]. The most essential primary benefit to a competent patient willing to take the medication could be the reminder to take the pill. However, this could also be achieved by a simple alarm [16]. With the progression of MS disease, a simple alarm will not be enough to ensure safety related to taking medications. The reasons for this are cognitive changes, communication problems, difficulty swallowing [17], and motor dysfunctions due to muscle weakness, abnormal walking mechanics, balance problems, spasticity, and fatigue [18]. The systematic administration of drugs and ongoing monitoring of drug therapy are crucial components of long-term care for patients with MS. Only proper medication management can help slow the course of the disease and manage symptoms. This result confirms the high level of awareness of the MS population about health problems and the conditions for effective treatment. Drug monitoring systems can reduce health-related costs in this patient group.

The cognitive support functionalities were important for the respondents, especially in regard to the questions about reminding them of meal or drink times (high priority). On the contrary, elderly people with cognitive impairment reported that these functionalities are not necessary to be implemented [11]. We hypothesize that older participants have different perceptions of disability. For them, the need to be reminded about basic human needs can be understood as admitting defeat [19]. The younger participants (MS population) lead a busier life, and it is normal for them to use memory strategies in their basic daily activities. About 65% of MS respondents declared using memory aids; in over 90% of cases, they were smartphones with calendars, alarms, and a task list.

Regarding the ability of the robotic assistant to provide cognitive exercise, it was highly important for medical staff, and of medium importance for the MS respondents. The lower importance was more likely to be chosen by younger participants with a lower level of disability (EDSS) who did not report cognitive difficulties.

It is worth mentioning that cognitive impairment may occur in patients with multiple sclerosis in the absence of other neurological signs or symptoms. This means that cognitive impairment can occur in patients with radiologically isolated syndrome, clinically isolated syndrome, or even the so-called benign form of multiple sclerosis [20]. Cognitive impairment is a common cause of job loss by MS patients [21]. Recent research has been increasingly focused on the cognitive system. Data increasingly shows that cognitive processing speed and memory are amenable to cognitive training interventions [20]. Thus, robotic assistance could be a novel strategy to improve cognitive functions and prevent cognitive decline.

In addition, the functionalities related to the performance of physical exercises (stimulation, delivery of instructions) were rated by MS respondents as medium priority, contrary to medical personnel, for whom they were high priority. The differences in prioritization can be partially explained by the fact that patients with MS frequently decrease physical activity due to the fear of worsening their symptoms [22]. There is now much evidence that an individualized exercise program can improve the physical and functional performance of MS patients [23]. This is reflected in the high priority given by medical staff.

Higher priority was associated with a higher level of intensity of experienced fatigue, whereas a higher level of intensity of experienced depression was associated with a lower priority of robot-assisted exercise. Exercise can result in a small, but important, reduction in fatigue among persons with MS [24]. Depression can increase any clinical sign in patients with MS (anxiety, pain, fatigue, and cognitive impairment), which may negatively affect the willingness to exercise [25].

The functionalities which were assigned medium-level priority concerned basic daily activities, such as preparing food, dressing, and finding and reaching for things to bring to patients. This result was demonstrated in previous research on the expectations of older people [11,26,27].

Verbal communication and using a touch screen were chosen as a high priority, and respondents indicated that a robotic assistant must be able to listen and respond to commands, as well as display information on a touch screen, which must be easily controlled. It is not surprising that respondents identified these ways in which a robotic assistant should be operated as high priority, as speech disorders and problems with coordination of movements are common in the MS population with the progression of the disease.

For the MS respondents, the way of interacting with the robotic assistant, concerning the possibility of expressing feelings by the robotic assistant, was not necessary to implement (low priority). A different opinion was expressed by the medical staff, who stated that the robotic assistant should be able to express emotions (medium priority). One-third of the psychologists surveyed explained why interaction through the expression of emotions through a robot assistant is important, namely, that it can have a positive effect in reducing emotional distress in users. Emotional distress is three times more common in people with MS than in the general population, which is associated with MS symptoms that occur in unexpected, unpredictable ways [28].

In terms of socio-emotional wellness, with a medium level of priority, our participants identified stimulating the patient to keep in touch with family and friends. Feelings of loneliness may negatively impact a person’s ability to act and engage in everyday activities. The robotic assistant can help people with health problems maintain a positive social life by supporting them in social interactions, and should not reduce or replace social interaction with people [29].

Another aspect of the expectations for the robot is its appearance. Kodate et al. identified, in a questionnaire study, that robots’ design (e.g., appearance, color, shape, materials) is one of the factors that respondents consider as important in their decision-making concerning home-care robots [30]. The overall appearance of the RAMCIP prototype had a high level of attractiveness in our study. The majority of respondents (over 95%) found the neutral appearance of RAMCIP very appropriate, and the height of the robotic assistant being shorter than the user was considered the best option for approximately 90% of respondents, which confirms findings from other studies [11,26]. The majority of MS respondents (about 90%) preferred the round face of the robotic assistant, and over 50% of MS respondents found that the robotic assistant should have a softer case than the RAMCIP presented.

In the overall context of the acceptance of social robots in our focus groups, we found a positive attitude towards home-care robots among participants. This study shows that MS patients and medical personnel found a good usability of the robotic assistant, as well as its high motivating potential, which may positively affect the quality of life of MS patients. It is highly likely that the way the material from the RAMCIP project was demonstrated to participants led to a positive perception of a robot assistant in most participants. Promoting the appropriate expectations for individuals unfamiliar with robotic healthcare may give the desired effect [31]. Rudigkeit and Gebhard (2020) also presented a positive attitude of a MS patient to an assistive human-robot (AMICUS 2) during an experimental interaction [32]. It is known that patients who find systems useless and non-motivational have more difficulty in their use [33]. According to the opinion of MS respondents, the assistant robot has a higher usability compared to all kinds of medical applications, with the proportion of responses 117/58 in favor of the robot. The proportion of responses in medical staff was 121/7 in favor of the assistant robot’s functionality. Since 2010, there has been an increase in the number of studies exploring assistive technologies, and many studies reported encouraging adoption rates and recommended further experimental studies [34,35,36,37,38].

One of the problems in introducing new technologies is the postponed willingness to cooperate with it among potential users. Among our respondents, deferred readiness can be observed, and the majority (over 70%) would accept a robot to help them gain independence when they lose the ability to function independently. Our findings are in line with previous studies where the participants reported a higher intention to use the robot in the future than at the present time [39]. The reason for this attitude may be the lack of familiarity with this type of technology, which causes uncertainty about the use of the robot [40].

The strength of our study is that we had experience in this field from a previous research project, RAMCIP, and we had material that helped familiarize our respondents with the topic. Additionally, the results are from mixed methods, and the number of participants from the two stakeholder groups was adequate to obtain reliable results. Several aspects must be considered while interpreting our study findings. The methodology used can be considered as promotional, presenting RAMCIP as a performance-enhancing product that enables a successful home and social life and increases independence. Such methodology may generate biased responses. On the other hand, unfamiliarity with assistive technology and a lack of an idea of its current capabilities may affect the feeling of uncertainty, and this may result in no response or passive responses, e.g., “I don’t know”.

## 5. Limitations

The limitation of this study is that it does not directly follow the real interaction of the participants with the assistant robot. The second limitation comes from the fact that the respondents came from one medical center. However, this weakness can be partially attenuated by the fact that people with MS come to this center from all over the country.

## 6. Conclusions

The results from our study might contribute to a better adaptation of robotic assistants to a broad range of needs and expectations of the MS population. The awareness gained from the COVID-19 pandemic must propel us to put into wider practice the use of assistive technologies that provide necessary care to many patients. Robotic assistance is designed to improve functioning, enable successful living at home and in the community, and enhance independence. To the best of our knowledge, most persons with MS will need different types of multidimensional assistance within the course of their disease progression, such as provision, education, instruction, and physical assistance. The provision of robotic assistance for persons with MS could potentially diminish activity limitations and participation restrictions; support cognitive function and positive attitude (depression prevention); reduce fatigue by energy conservation; and, ultimately, improve quality of life.

## Figures and Tables

**Table 1:** Demographics and disease characteristics of the MS study group.

Variables	
Age [n (%)]	
21–30	30 (17.05%)
31–40	44 (25.00%)
41–50	45 (25.57%)
51–60	36 (20.45%)
60+	21 (11.93%)
Female/Male [n (%)]	132 (75%)/44 (25%)
Education level [n (%)]	
Elementary	10 (5.68%)
Vocational	27 (15.34%)
Secondary	62 (35.23%)
Higher	77 (43.75%)
Employment status [n (%)]	
Self-employment	13 (7.39%)
Full-time employee	47 (26.70%)
Student	6 (3.41%)
Retired	95 (53.98%)
Unemployed	13 (8.52%)

**Table 2:** Demographic and disease characteristics of the MS study group.

Course of disease [n (%)]	
Relapsing remitting	114 (64.77%)
Secondry progressive	30 (17.05%)
Primary progressive	25 (14.20%)
Progressive relapsing	7 (3.98%)
Duration of the disease [n (%)]	
<1 year	6 (3.41%)
1–2 years	9 (5.11%)
2–5 years	25 (14.20%)
5–10 years	43 (24.43%)
>10 years	93 (52.84%)
Level of disability (EDSS) [n (%)]	
0–4.0	47.73%
4.5–6.5	37.50%
7.0–10.0	14.77%
Depression [n (%)]	
never	28 (15.91%)
hardly ever	34 (19.32%)
sometimes	73 (41.48%)
often	38 (21.59%)
almost always	3 (1.70%)
Intensity level of depression [n (%)]	
1–5	104 (59.09%)
6–10	72 (40.91%)
Cognitive problems [n (%)]	
never	31 (17.61%)
hardly ever	25 (14.20%)
sometimes	67 (38.07%)
often	47 (26.70%)
almost always	6 (3.41%)
Intensity level of memory and concentration problems [n (%)]	
1–5	110 (62.50%)
6–10	66 (37.50%)
Fatigue [n (%)]	
never	5 (2.84%)
hardly ever	13 (7.39%)
sometimes	58 (32.95%)
often	70 (39.77%)
almost always	30 (17.05%)
Fatigue level [n (%)]	
1–5	63 (35.78%)
6–10	113 (64.22%)

EDSS—Expanded Disability Status Scale.

**Table 3:** Prioritization of target use cases of the robotic assistant.

**A. Safety**				
**Safety**	**Mean**	**Users Priority**	**Mean**	**Medical Staff Priority**
Detection of falls	1,668,966	H	1,456,311	H
Asks the patient how they feel after falls	2,055,172	M	1,968,452	H
Informs family members about unwanted incident at home	1,506,849	H	1,494,624	H
Calls for help if something happens to the patient or dangerous situations at home are detected (detects smoke or gas)	1,621,622	H	1,359,223	H
Detection of obstacles on the floor to prevent falls	1,932,432	H	1,902,174	H
Turns the light on when it is too dark and the person starts moving around the house	1,956,989	H	1,902,913	H
Recognizes when it can or cannot open the house door	2,652,778	M	2,516,129	M
Turns working home appliances (electric, water, gas) off while user is busy and asks to do it	1,768,707	H	188,172	H
Monitors correctness of the patient’s medication intake	1,978,521	H	1,556,732	H
Controls proper daily amount of water	1,841,096	H	1,734,750	H
Keeps alert at night	1,643,466	H	1,525,592	H
**B. Cognitive Aids**				
**Cognitive Aids**	**Mean**	**Users Priority**	**Mean**	**Medical Staff Priority**
Provides cognitive exercise to the patient	2,110345	M	1,932,432	H
Reminds the patient that it is time for them to take their medication	1,958621	H	161,165	H
Reminds about regular water drinking and meal time	1,956989	H	1,980,583	H
Reminds about important dates (e.g., medical appointments, events, deadlines)	2,041096	M	227,957	M
**C. Physical Aids**				
**Physical Aids**	**Mean**	**Users Priority**	**Mean**	**Medical Staff Priority**
Stimulates/provides instructions to the patient to perform physical exercises	2,204,082	M	1,979,452	H
Can reach medication which is difficult to reach for the patient	2,158,621	M	2,086,022	M
Reaches for fallen utensils and hands them over to the patient to prevent the patient from bending over. Grasps things from the floor/shelves	2,296,552	M	2,106,796	M
Brings food	2,555,556	M	2,451,613	M
Helps the patient take on/off her/his shoes	2,643,357	M	2,361,702	M
Finds the things the patient is looking for	2,482,759	M	2,445,652	M
Brings the things the patient asks for	2,423,611	M	2,326,087	M
Helps the patient properly button her/his clothes	2,671,329	M	2,322,581	M
**D. Household Management and Socio-Emotional Wellness**				
**Household Management**	**Mean**	**Users Priority**	**Mean**	**Medical Staff Priority**
Helps the patient clean the house	2,421,769	M	2,698,925	M
Helps the patient with a shopping list	3,048,611	L	2,923,913	M
Helps the patient prepare food	2,643,836	M	2,673,913	M
Detects an open fridge door and closes it	2,503,448	M	2,300,971	M
Stimulates the patient to keep in touch with family and friends	2,393,103	M	2,408,602	M

H—High priority; M—Medium priority; L—Low priority.

**Table 4:** Prioritization of different capabilities of the robotic assistant in relation to human interaction.

Communication and Interaction	Mean	Users Priority	Mean	Medical Staff Priority
The robotic assistant can reply to simple questions (e.g., what time is it?)	2,372,414	M	2,225,806	M
The robotic assistant can listen and respond to simple commands you give	1,979,452	H	1,882,796	H
The robotic assistant can comprehend and respond to simple gestures you make	2,263,889	M	2,107,527	M
The robotic assistant can take part in dialogue interactions with the user to complete required tasks	2,472,603	M	2,397,849	M
The robotic assistant can talk to you regarding its current task/state	2,331,034	M	2,301,075	M
The robotic assistant can be easily controlled by the touch screen which is mounted on it	1,896,552	H	1,956,989	H
The controls shown on the touch screen of the robotic assistant change to reflect the needs of the user and the current task	2,082,192	M	2,043,011	M
The robotic assistant can display information on a touch screen that is mounted on it	1,968,966	H	1,902,174	H
The robotic assistant can be controlled directly through the touch screen it carries without the need to engage in a dialogue with the user	2,294,521	M	2,150,538	M
The robotic assistant has a face that can express its feelings throughout interactions with the user	3,027,397	L	2,602,151	M
The robotic assistant should continuously listen to the user for commands	2,184,932	M	2,139,785	M
The robotic assistant can understand the psychological state of the user and provide positive affective impact (actions)	2,458,333	M	2,391,304	M

H—High priority; M—Medium priority; L—Low priority.

**Table 5:** Correlates of demographics, disease-specific variables, and priority level of the functionalities.

	**Provision Cognitive Excersises**			
	**Mean**	**SD**	**95% CI**	***p*** **-Value**
Age	45	15	±2.07	<0.001
EDSS:				
0–4.0	2.56	1.02	±0.214	NS
4.5–6.5	5.61	0.83	±0.00	<0.001
7–10	8.46	0.97	-	NS
Cognitive problems	4.35	2.52	±0.295	<0.001
	**Performance of Physical Excersises**			
	**Mean**	**SD**	**95% CI**	***p*** **-Value**
Fatigue	6.14	2.48	±0.293	<0.001
Depression	4.60	2.70	±0.295	<0.001

SD—Standard Deviation; CI—Confidence Interval; NS—Not Significant.

## Data Availability

The data presented in this study are available on request from the corresponding author. The data are not publicly available because data supporting reported results do not exist in electronic version.
